# Controlled Release of Simvastatin from Biomimetic β-TCP Drug Delivery System

**DOI:** 10.1371/journal.pone.0054676

**Published:** 2013-01-18

**Authors:** Joshua Chou, Tomoko Ito, David Bishop, Makoto Otsuka, Besim Ben-Nissan, Bruce Milthorpe

**Affiliations:** 1 Advanced Tissue Regeneration and Drug Delivery Group, School of Medical and Molecular Sciences, University of Technology Sydney, Sydney, New South Wales, Australia; 2 Faculty of Pharmacy, Research Institute of Pharmaceutical Science, Musashino University, Nishi-Tokyo, Japan; 3 School of Chemistry and Forensic Sciences, University of Technology Sydney, Sydney, New South Wales, Australia; 4 Faculty of Science, University of Technology Sydney,Sydney, New South Wales, Australia; National University of Ireland - Galway, Ireland

## Abstract

Simvastatin have been shown to induce bone formation and there is currently a urgent need to develop an appropriate delivery system to sustain the release of the drug to increase therapeutic efficacy whilst reducing side effects. In this study, a novel drug delivery system for simvastatin by means of hydrothermally converting marine exoskeletons to biocompatible beta-tricalcium phosphate was investigated. Furthermore, the release of simvastatin was controlled by the addition of an outer apatite coating layer. The samples were characterized by x-ray diffraction analysis, fourier transform infrared spectroscopy, scanning electron microscopy and mass spectroscopy confirming the conversion process. The *in-vitro* dissolution of key chemical compositional elements and the release of simvastatin were measured in simulated body fluid solution showing controlled release with reduction of approximately 25% compared with un-coated samples. This study shows the potential applications of marine structures as a drug delivery system for simvastatin.

## Introduction 

In the continual development of drug delivery systems for bone tissue engineering it is widely accepted that the efficiencies of such system can be further improved by “*controlling*” or “*slowing*” the release of pharmaceutical compounds enabling prolonged therapeutic effect. The primary goal of such delivery systems in bone tissue engineering is to stimulate bone growth in the local environment at the site of interest and many promising systems have been developed over the years. Compounds such as bone morphogenetic proteins, growth factors, and pharmaceutical compounds are commonly incorporated in drug delivery systems. In recent years, particular interest in the use of statins [3-hydroxy-3 methylglutaryl coenzyme A (HMG-CoA reductase) inhibitors], which are used as a cholesterol-lowering drugs have shown promising effectiveness in a wide range of medical applications. One of the key benefits on the use of statins is its ability to increase the expression of bone morphogenetic protein-2 (BMP-2) and vascular endothelial growth factor (VEGF) [1]–[3]. These studies suggest they can be beneficial in the treatment of osteoporosis, fractures, and bone defects. Studies have been carried out to investigate the effects of systemic administration on bone healing [4]–[7], oral ingestion [4], [8] and in local applications on bone repair [9]–[12]. Despite the obvious benefits of statins, there are some associated side effects. It has been found that exceedingly high dosage of statins applied systemically can increase the risk of liver failure, kidney disease, and rhabdomyolysis [13]. While low dosages can be inefficient in bone healing, higher doses can stimulate subsequent inflammation [14]. As such this study seeks to develop a controlled release delivery system capable of delivering simvastatin.

Whilst most attention has been in developing delivery vehicles by conventional synthetic materials, biomimetic structures have been mostly overlooked. Fossilized coral exoskeletons naturally possess uniform and interconnected porous network capable of allowing more effective and predictable drug loading. Furthermore, as these exoskeletons are chemically composed of calcium carbonate, which has a faster dissociating rate, can be easily converted to calcium phosphate derivatives whilst retaining the original structural integrity. This will allow us the ability to control the degradation rate of the delivery vehicle. In addition, calcium phosphates are biocompatible and as part of its natural degradation process, calcium ions will be released which can provide additional supplement in the healing of bone repair. This study looks at evaluating and characterizing coral derived beta-tricalcium phosphate (β-TCP) as a drug delivery scaffold material for simvastatin delivery.

## Materials and Methods

### Hydrothermal Synthesis of β-TCP Stars

*Foraminifera* samples were purchased commercially from Business Support Okinawa Co. Ltd., Japan. The samples were first cleansed in sodium hydrochlorite for 20 mins and dried at 40°C for 2 hours and placed in a heating oven at 220°C for 48 hours with aqueous diammonium hydrogen phosphate [(NH_4_)_2_(HPO_4_)] (Wako Chemical Co., Tokyo, Japan). The diammonium hydrogen phosphate solution was adjusted to yield Ca/P molar ratios of 1.5 to produce β-TCP. The resulting samples were than subsequently characterized by the following methods. Furthermore no specific permits were required for the described field studies.

### Physico-chemical Characterization

The powder X-ray diffraction (XRD) profiles of the coral before and after hydrothermal conversion were measured by powder XRD analysis (RINT- Ultima-III, Rigaku Co., Japan; CuKα radiation, 40 kV, 40 mA). The step scanning was performed with an integration time of 1 min at intervals of 2° (2θ) and matched with JCPDS database. The chemical composition of the crushed sample powder was investigated by fourier transform infrared spectroscopy (FTIR). Samples were ground with 1% KBr in an agate mortar, and analyzed under nitrogen atmosphere from 2000 to 400 cm^−1^ using a Nicolet IR 760. Inductively coupled plasma-mass spectroscopy (ICP-MS) was used to measure the chemical composition of the samples by using approximately 0.3 g of sample which was digested with 0.25 mL of HNO_3_ and 0.25 mL of H_2_O_2_. Once the digestion was completed the sample volume was made up to 5 mL with H_2_O. The samples underwent a further 1∶100 dilution before ICP-MS analysis. Samples were diluted further as needed**.** The surface morphology was characterized by scanning electron microscopy (JEOL JSM-7600F, Field Emission SEM, 10 KV). The internal architectural structure was characterized by a micro-CT scanner (InspeXio; Shimadzu Science East Corporation, Tokyo, Japan) with a voxel size of 70 mm/pixel as a non-destructive method. Tri/3D-Bon software (RATOC System Engineering Co. Ltd, Tokyo, Japan) was used to generate a complete 3D reconstruction of the sample. The surface area was measured by using a Quantachrome Monosorb™ B.E.T. surface area analyzer and the pore size distribution profile was measured by nitrogen volumetric adsorption measurements (Quantachrome Autosorb pore size analyser).

### Evaluation of *in-vitro* Degradation of β-TCP

The *in-vitro* degradation of the β-TCP looking specifically at the release of calcium and magnesium was evaluated in simulated body fluid solution (SBF) [15]. The samples were each immersed in 5 mL of the buffer solution and placed in a shaking water bath at 37°C. At each predetermined time point, the buffer solution were collected and replaced with fresh buffer every 24 hours for 7 days. The collected solutions were than evaluated by ICP-MS.

### Production of Simvastatin Drug Delivery System

Simvastatin solution (Watanabe Chemical Co., Osaka, Japan) at a concentration of 4 mg/mL were immersed with the β-TCP samples in a rotaevaporator (Buchi Rotavapor RT200) until the solution were dried and subsequently placed in a 100% humidity vacuum seal. The simvastatin loaded β-TCP were further coated with an apatite outer layer. The apatite cement bulk powder consist of equimolar mixture of tetratricalcium phosphate (TTCP) and dicalcium phosphate dihydrate (DCPD) (Wako, Tokyo) and was prepared by grinding at 20 per second for 17 mins in an agate vibration mixer mill (Retsch Co., Germany; 10 mL volume chamber in a ball 10 mm in diameter). This cement bulk powder (0.470 g) was poured into a silicon rubber mould (5 mm in diameter with 2 mm thickness) for 1 hour, and stored at room temperature in a vacuum seal with 100% relative humidity for 24 hrs.

### *In-vitro* Evaluation on the Release of Simvastatin

The amount of simvastatin released in SBF solution was measured by UV-spectroscopy (Shimadzu UV-2550 Spectrophotometer) at 238.5 nm wavelength and the concentration was calculated with reference to standards prepared fresh for each analysis. A shaking water bath (Personal-11, TAITEC Co., Japan) was used for the dissolution release study. The solvent content was 5 mL for each buffer solution, and replaced with fresh 6 mL of each respective solution at 2, 4, 6, 8, 24, 48, 72, 96, 120, 144, 168 and 192 hours. The temperature of the dissolution test was conducted under 37°C with a shaking rate of 180 times/min. SBF solution was prepared according to Kokubo’s method [15].

### Statistical Analysis

All data were examined based on 5 different measurement values and presented as standard deviation. Repeated measurement analysis of variance (ANOVA) was used to determine significant differences among the groups and a p-value of 0.05 was considered significant.

## Results

### Physico-chemical Characterization of β-TCP Stars

**Figure 1:**
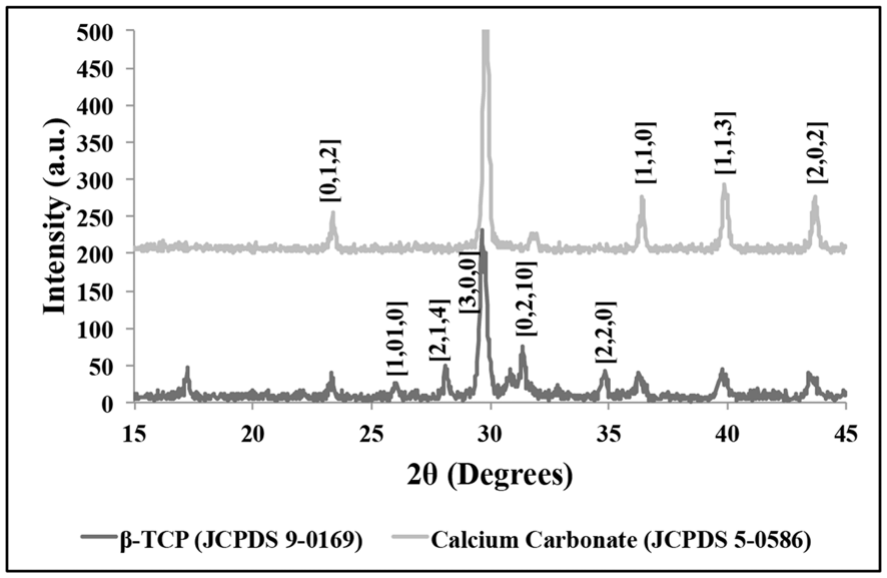
XRD spectra showing before and after hydrothermal conversion of sample with matching peaks corresponding to calcium carbonate and β-TCP.

**Figure 2:**
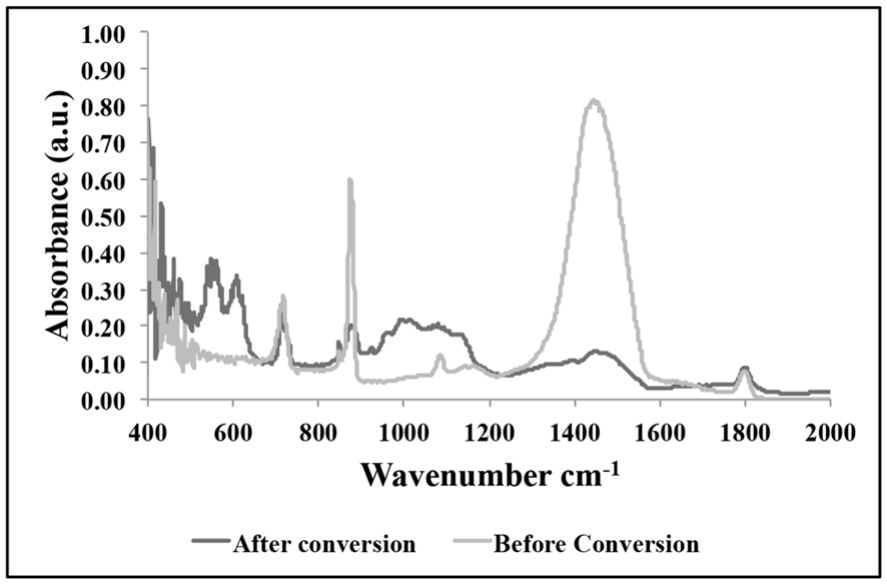
FTIR spectra comparing before and after hydrothermal conversion reinforcing conversion of calcium carbonate sample to β-TCP.

The goal of this research is to determine if coral exoskeletons can be hydrothermally converted to β-TCP and be used as a drug delivery system for simvastatin with the aim of stimulating faster and better bone regeneration. The first of the series of analysis involves characterizing the samples before and after conversion to confirm if the conversion took place and to evaluate any significant changes in the physical and chemical properties of the material. Figure 1 shows the results from XRD analysis and shows matching peaks from JCPDS database associated with calcium carbonate (before conversion) and β-TCP (after conversion). There remains a small amount of carbonate peaks after conversion but this will only affect the dissolution rate of the material and does not in any way affect the outcome of the goal of this research. FTIR pattern shown in Figure 2 provides additional supporting data showing the carbonate bands ν_2_ at 866 cm^−1^ (labile carbonate group) and ν_3_ at 1420 cm^−1^. The after conversion pattern shows the phosphate band ν_3_ and ν_4_ in the ranges 1120 cm^−1^ to 1000 cm^−1^ and 670 cm^−1^ to 530 cm^−1^. The band at 865 cm^−1^ corresponds to the P-OH stretching mode of HPO_4_ groups and the small peak at 1454 cm^−1^ to 1414 cm^−1^ is from the C-O of CO_3_ groups which indicate the presence of small amount of remaining carbonate in the material. Furthermore, an in-depth chemical compositional analysis of the samples was performed by mass spectroscopy and the results are presented in Table 1.

**Table 1:** Compositional make-up of samples before and after hydrothermal conversion.

Elements	Before Conversion (x10^4^ ppm)	After Conversion (x10^4^ ppm)
Calcium	1.69	1.68
Phosphate	0.02	6.21
Strontium	0.18	0.21
Magnesium	3.06	3.08

**Figure 3:**
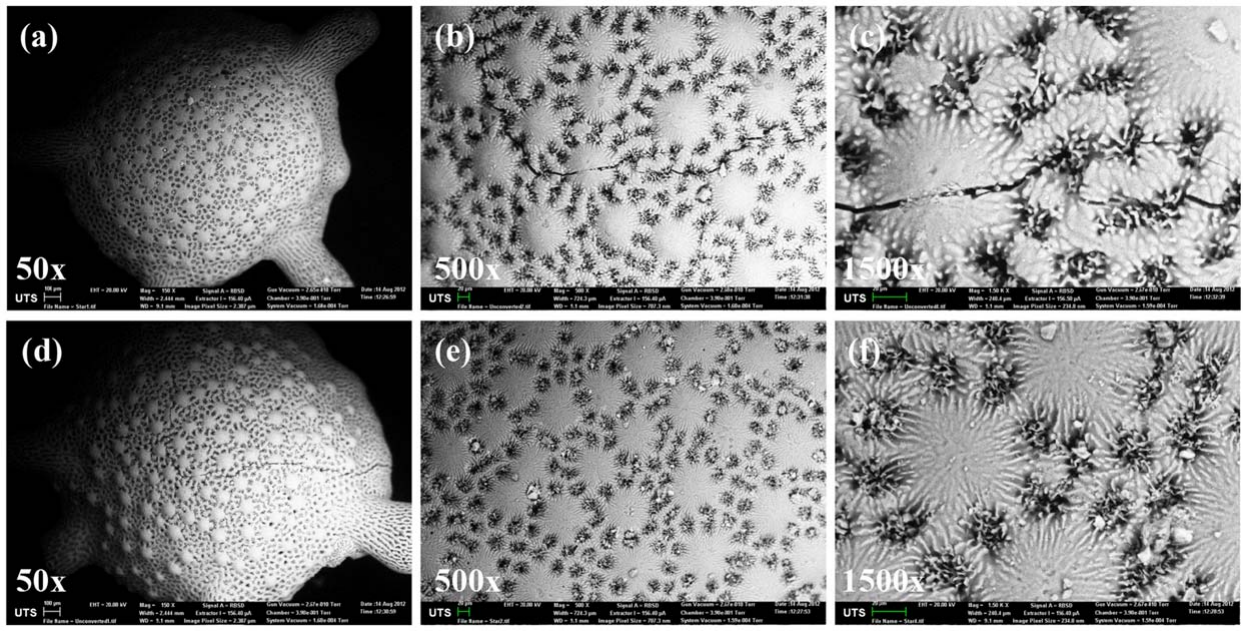
SEM images showing preservation of the morphological and structural integrity of the sample before conversion (a,b and c) and after conversion (d, e and f). The images also detail the uniform distribution of surface pores.

**Figure 4:**
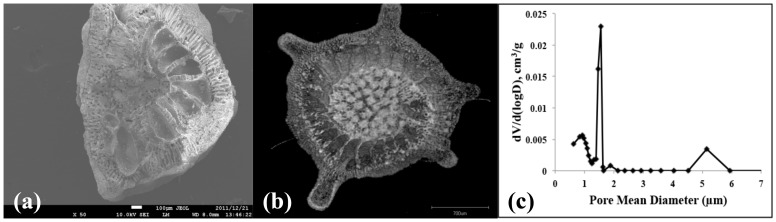
Cross sectional image taken by SEM showing various porous chambers within the material (a); micro-CT image showing similar structure with a denser porous network towards the center of the material and the pore size distribution profile of the material is shown in (c).

The amount of calcium before and after conversion remained at a similar amount and the presence of phosphate is clearly observed. The amount of strontium and magnesium was also preserved during the conversion process. The morphological characterization was observed by scanning electron microscopy and Figure 3 (a, b and c) shows the coral exoskeleton before conversion and Figure 3 (d, e and f) shows the material after hydrothermal conversion. It is clear that there is no significant morphological change during the conversion process and the architectural structure and integrity of the material was preserved during this process. Furthermore, the higher magnification images shows uniform pore distribution across the surface of the material thereby allowing potential increase drug loading efficiency. This again highlights the advantages of biomimetic materials combined with hydrothermal conversion technique. The above results therefore confirm that the coral materials were successfully converted to β-TCP. The internal structure of the coral exoskeleton was examined by examining the cross sectional cut of the sample and observed by SEM (Figure 4 (a)) and micro-CT (Figure 4 (b)) analysis. SEM imaging showed that the exoskeleton is internally compartmentalized with various “chambers” interconnected by pores beneath the surface of the material. Micro-CT analysis was used as a non-destructive method to study the internal architectural structure. The image again shows that porous chambers further connected near the center of the structure with a much denser porous network. These compartments of porous chambers and interconnected pores are truly unique and so far irreproducible synthetically whilst offering an ideal structure for various biomedical applications such as drug delivery system. The surface area of the material was measured to be 0.138±0.004 m^2^/g. The corresponding pore size distribution profile of the sample is presented in Figure 4 (c) showing the presence of larger pores ∼5 µm with the majority of the pore size ∼1.5 µm. The distribution profile also indicate the presence of <1 µm pores.

### Characterization of β-TCP Drug Delivery System

**Figure 5:**
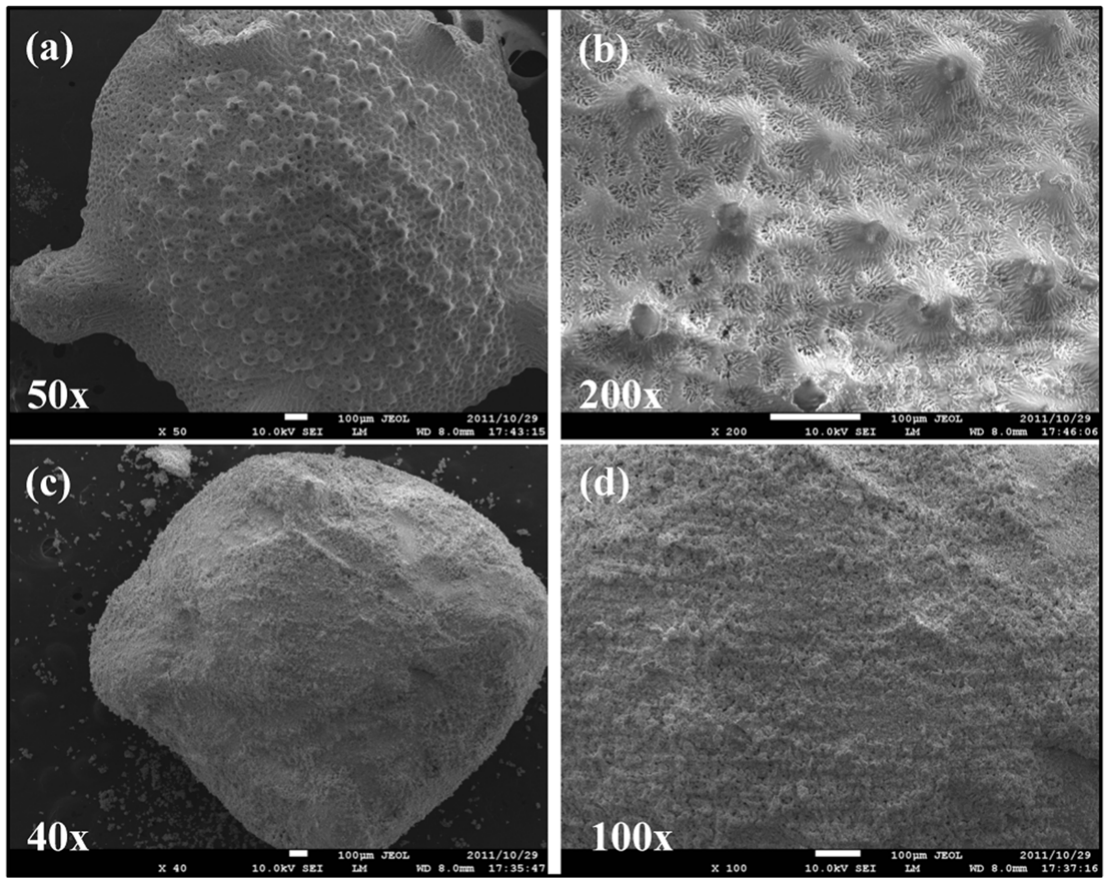
SEM images showing simvastatin loaded β-TCP sample [(a), (b)] and with apatite coating [(c), (d)] showing a spherical coating around the sample material.

The load concentration of simvastatin in the β-TCP material was calculated to be 23 µg/mg and with the apatite coating this was found to be 13 µg/mg. There is some loss of simvastatin during the production of the apatite coated β-TCP system but this is not at a significant amount. The morphological observation was done by SEM in Figure 5 where (a) and (b) shows the β-TCP stars with simvastatin and (c) and (d) shows simvastatin incorporated with the apatite coating. The method for the apatite coating is easily reproducible and can produce consistent and uniform coating around the material.

### Comparative in-vitro Dissolution Profile of β-TCP Stars

**Figure 6:**
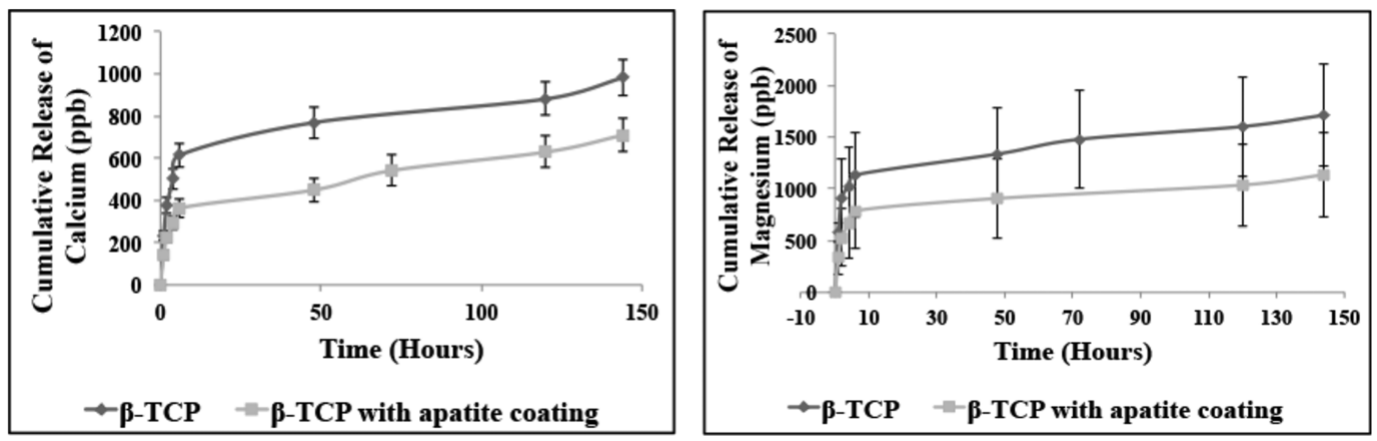
The *in-vitro* dissolution profile in SBF solution showing the release of (a) calcium and (b) magnesium with and without the apatite coating. The result shows slower release of both ions with the apatite coating.

As the scaffold material is intended for use as a prolonged drug delivery system, it is vital to evaluate the dissolution profile of the β-TCP stars and to ascertain if the apatite coating had any effect on dissolution. This study was examined *in-vitro* by immersion in SBF solution which mimics closely to the physiological environment. Figure 6 (a) shows the cumulative release of calcium with and without the additional apatite coating over a 7 day period. The amount of calcium released is within the ppb range and it can be seen that with the additional apatite coating that the release of calcium has been “*slowed down*” while retaining the same release pattern. Figure 6 (b) shows the release profile of magnesium which shows a similar trend and release pattern before with and without the apatite coating. The release of strontium is not presented as there was no detected release of strontium over the 7 day experimental period which implies that the release of strontium would be delayed to a later stage of the dissolution process.

### In-vitro Release of Simvastatin from β-TCP Stars

**Figure 7:**
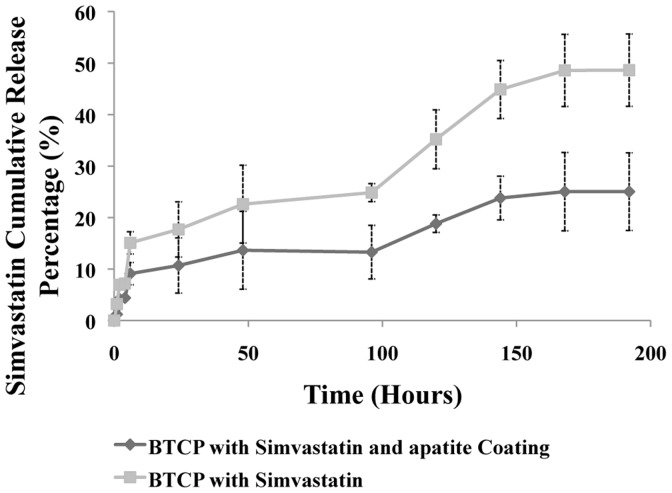
The *in-vitro* release profile of simvastatin with and without the apatite coating showing controlled and slower release of the drug by 20% over 7 days.

Figure 7 shows the *in-vitro* percentage release of simvastatin in SBF solution over 7 days comparing apatite coated β-TCP stars and non-coated samples. From the results the non-coated samples released an average of 48% over the 7 days where the apatite coated group only released an average of 21% over the 7 days. It is clear from the result that with the apatite coating the release of simvastatin was delayed by approximately 20%.

## Discussion

Studies have shown the potential effect of simvastatin as an active promoter of BMP-2 gene markers and its active role in the complex mechanism of bone reconstruction. The overall goal of the present study was to investigate whether marine exoskeletons can be used as potential carrier for the delivery of simvastatin. It is of great importance to develop inexpensive and strategies of producing calcium phosphate synthesis methods focused on the precise control of morphology and chemical composition. Because the hydrothermal conversion reactions are solution-mediated, the composition can be controlled. Our approach includes hydrothermally converting the material to biocompatible β-TCP combined with an outer apatite coating to slow the release of simvastatin. The physico-chemical analysis confirms the successful conversion from calcium carbonate coral exoskeleton to β-TCP though there remains a small quantity of carbonate but this will only slightly affect the overall dissolution rate of the bulk material. One of the key advantages of the hydrothermal conversion is the preservation of the original architectural structure of the marine exoskeleton. This is crucial as the uniform pore distribution combined with the interconnected porous network provides the appropriate setting for higher efficiency drug loading and more predictable release. This can be observed from both the degradation of calcium, magnesium and the release of simvastatin. The results showed that with the apatite coating the release of key compositional elements and simvastatin was slowed down while maintaining similar release pattern compared without the coating. Furthermore, the release of simvastatin was reduced by approximately 20% which is significant for prolonged delivery of the drug. This will limit the initial burst release and provide the time for the inflammation around the surgical site to subside and allow the continual delivery of simvastatin. It is also important to note the significance of the natural degradation of the β-TCP stars. Apart from the release of simvastatin from the material, calcium ions are also being released with the addition of magnesium which studies have shown to benefit in the formation of healthier and stronger bones [16]. In addition, even though in our 7 day release study the release of strontium was not detected, from the compositional analysis strontium is present in the material and many studies have shown the potential benefits of strontium in the bone regeneration and healing process [17]. As such, these results presents a dynamic delivery system where key bioinorganics in conjunction with pharmaceutical compound are combined together in hope of providing an effective therapy for bone related problems. The envisioned goal of this research is the development of a sustained delivery of simvastatin as a systemic therapy for the treatment of osteoporosis or bone fracture as current delivery systems for simvastatin are still limited in their therapeutic efficiencies. The intended and proposed route of administration of the simvastatin loaded β-TCP delivery system is to implant at either the local site of trauma or in the intramuscular region near the affected site. The rationale for this is to provide a localized delivery of simvastatin to the affect or trauma site. Intramuscular implantation can help with the systemic delivery of simvastatin via the local blood vessels. This work will be the subject of future experiment.

### Conclusion

In conclusion, this research showed that marine exoskeleton with appropriate morphology and structures can be incorporated with simvastatin and its release can be controlled with an outer apatite coating. This will enable a more prolonged therapeutic delivery of simvastatin allowing the drug to sustain its effect in the repair and healing of bone related fractures.
